# Skin Biophysical Characteristics in Patients with Keratoconus: A Controlled Study

**DOI:** 10.1155/2016/6789081

**Published:** 2016-06-15

**Authors:** Reza M. Robati, Bahram Einollahi, Hoda Einollahi, Shima Younespour, Shahed Fadaifard

**Affiliations:** ^1^Skin Research Center, Shahid Beheshti University of Medical Sciences, Tehran, Iran; ^2^Department of Ophthalmology, Shahid Beheshti University of Medical Sciences, Tehran, Iran; ^3^Department of Ophthalmology, Tehran University of Medical Sciences, Tehran, Iran

## Abstract

*Background*. Keratoconus is a relatively common corneal disease causing significant visual disability. Individuals with connective tissue disorders that affect the skin such as Marfan's syndrome and Ehlers-Danlos syndrome or patients with atopic dermatitis show an increased prevalence of keratoconus. It seems that there are some concurrent alterations of skin and cornea in patients with keratoconus.* Objective*. We plan to compare skin biophysical characteristics in patients with keratoconus and healthy controls.* Methods*. Forty patients with keratoconus (18 females and 22 males) with mean (SD) age of 33.32 (9.55) years (range 19–56) and 40 healthy controls were recruited to this study. Skin biophysical characteristics including cutaneous resonance running time (CRRT), stratum corneum hydration, and melanin values were measured in patients and controls.* Results*. The median CRRT, stratum corneum hydration, and melanin measurements were significantly lower in patients with keratoconus in comparison with healthy controls.* Conclusion*. There are some alterations of skin biophysical properties in patients with keratoconus. Therefore, the assessment of these skin parameters could provide us some clues to the possible common biophysical variations of cornea and skin tissue in diseases such as keratoconus.

## 1. Introduction

Cornea is a complex anisotropic composite with nonlinear elastic and viscoelastic properties. The corneal stroma makes up 90% of corneal thickness and is the main contributor to the cornea's strength and transparency. This layer is composed of 250–400 stacked lamellae. In the anterior one-third of the stroma, the lamellae are more narrowly interwoven than in the posterior two-thirds. The lamellae are composed of collagen fibrils oriented in specific directions [1]. Keratoconus (KCN) is a relatively common corneal disease causing significant visual disability. It is characterized by progressive irregular myopic astigmatism with central corneal thinning and protrusion. The disease usually manifests itself during the late teens or early twenties and shows a slow progression for the next decade or two. KCN is a multifactorial disease involving complex interaction of both genetic and environmental factors that contribute to the disease manifestation [2]. The preferred orthogonal fibril orientation of the normal cornea appears to be altered in KCN [3].

Individuals with connective tissue disorders that affect the skin such as Marfan's syndrome or Ehlers-Danlos syndrome and mitral valve prolapse show an increased prevalence of keratoconus [4–6]. Moreover, some associations have been reported between atopic dermatitis and keratoconus [7]. Regarding these data, it seems that there might be some links between cornea and skin biophysical characteristics. Therefore, we plan to evaluate the biophysical properties of skin including stratum corneum hydration, cutaneous resonance running time, and melanin value in patients with keratoconus and compare them with healthy controls.

## 2. Patients and Methods

Forty patients with keratoconus and 40 healthy controls frequency-matched for age and sex were enrolled in this case-control study. The study followed the tenets of the Declaration of Helsinki and was approved by the ethics committee of Shahid Beheshti University of Medical Sciences. Written informed consent was obtained from all of the participants.

KCN was diagnosed by an ophthalmologist, specialist in cornea. Patients were evaluated by slit lamp and topography. Keratoconus severity was classified as mild, moderate, severe, and very severe according to the mentioned ophthalmologic examination [8]. Patients who have ocular disease except for KCN, use any topical medication for the eye or skin disease, take any systemic drugs and contraceptives, have any dermatologic disease, smoke, and consume alcohol were excluded from the study. Controls who have any ocular disease, use any topical or systemic medication for the eye or skin disease, have any dermatologic disease, smoke, and consume alcohol were also excluded from the study.

The biophysical properties of skin were evaluated by Multi Probe Adaptor System (MPA 9, Courage and Khazaka Electronic GmbH, Köln, Germany) in both patient and control groups. The biophysical characteristics of skin were measured at two different locations of the body (the upper inner arm and neck). No skin care products were applied to the measured sites 24 hours prior to the measurement, and the measured sites were not washed with soaps or surfactants for at least 2 hours prior to the assessments. All the subjects remained inactive at 24–26°C, at a relative humidity of 50–55% for 30 minutes before the measurements were taken.

We measured cutaneous resonance running time (CRRT) with Reviscometer® RVM 600 (Courage and Khazaka Electronic GmbH, Köln, Germany) that allows for the evaluation of the mechanical properties of skin by measuring the propagation time of a shear wave between two sensors placed on the skin surface; one is transmitting an acoustical shockwave, and the other is the receiver. The time the wave needs to propagate from transmitter to recipient is the measured parameter that is defined as cutaneous resonance running time (CRRT). It determines the mechanical properties of the skin and the direction of collagen and elastic fibers. The CRRT is expressed in arbitrary units (AU). CRRT is mainly influenced by collagen fibers in the papillary layers of dermis and correlates negatively with skin stiffness. Directional changes of CRRTs during lifetime, which could reflect the dermal biophysical property at various ages, have been reported in some studies [9, 10]. We assessed CRRTs at various directions on the upper inner arm and neck.

Stratum corneum hydration was evaluated with a Corneometer® CM825 (Courage and Khazaka Electronic GmbH, Köln, Germany) that measures electrical capacitance of the skin as a reflection of water content of the horny layer. Melanin values of skin were evaluated by Mexameter® MX 18 (Courage and Khazaka Electronic GmbH, Köln, Germany). It measures the melanin and hemoglobin (erythema) of skin by reflectance.

### 2.1. Statistical Methods

The statistical software SPSS 16.0.0 (SPSS Inc. Chicago, IL, USA) was used for all data analyses. *p* values less than 0.05 were considered statistically significant. All tests were two-sided. Categorical data are expressed as number (percentage). Continuous variables are reported as mean (SD) or as medians with total and interquartile range (25th, 75th percentiles). The normality assumption of the continuous variables was examined using Shapiro-Wilk's *W*-test. Cutaneous resonance running time, stratum corneum hydration, and melanin measurements of patients with keratoconus and healthy controls were compared by the nonparametric Wilcoxon signed-rank test. In patients with keratoconus, the associations between both time since diagnosis and severity of disease and other continuous variables were assessed using Spearman's correlation tests.

## 3. Results

Forty patients with keratoconus (18 females and 22 males) with mean (SD) age of 33.32 (9.55) years (range 19–56) and 40 healthy controls were recruited to this study. Patients and controls were individually matched on age and gender. Eight patients had mild keratoconus (20%), 17 (42.5%) moderate keratoconus, and 13 (32.5%) severe keratoconus and two patients (5%) had very severe keratoconus. The median time to diagnosis was 5.75 years (range 3 months to 22 years).

The median CRRT measured by reviscometer on the neck and arm areas was significantly lower in patients with keratoconus than healthy controls (both *p* < 0.0001 and Table 1). Also, the median stratum corneum hydration and melanin measurements were significantly lower in patients with keratoconus in comparison with healthy controls (*p* < 0.0001, and *p* = 0.02, resp., and Table 1). There was no significant association between severity of keratoconus and CRRT on the neck (rs = 0.03, *p* = 0.84), CRRT on the arm (rs = −0.28, *p* = 0.07), stratum corneum hydration (rs = −0.22, *p* = 0.17), and melanin values (rs = −0.14, *p* = 0.39). In addition, no significant association was found between the time since diagnosis and CRRT on the neck (rs = 0.05, *p* = 0.78), CRRT on the arm (rs = −0.25, *p* = 0.12), stratum corneum hydration (rs = −0.01, *p* = 0.95), and melanin measurements.

**Figure 1:**
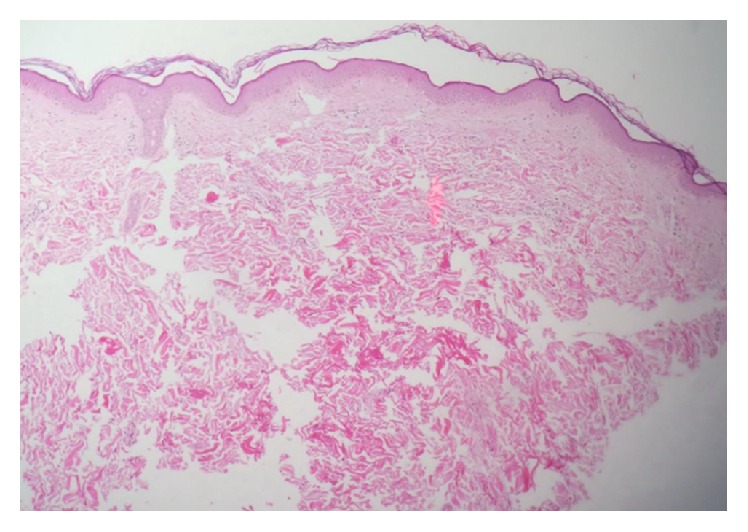
Histopathology view: skin of inner arm shows normal skin structures (H&E stains *∗*10).

**Figure 2:**
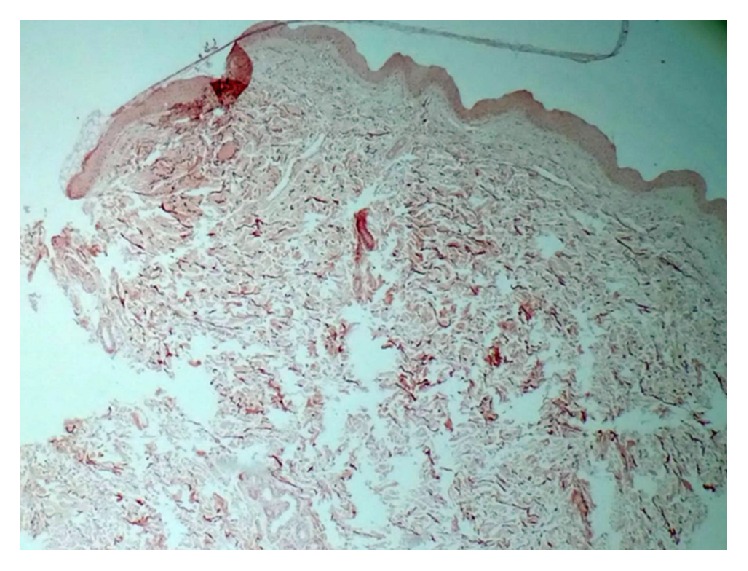
Histopathology view: decreased elastin content, particularly in mid-dermis (Acid Orcein-Giemsa *∗*10).

Five patients agreed to undergo a skin biopsy on the upper inner arm. Special staining with Acid Orcein-Giemsa was done. Three samples showed decreased elastin in dermis, especially in upper and mid-dermis (Figures 1 and 2).

## 4. Discussion

Keratoconus is a multifactorial disease involving complex interaction of both genetic and environmental factors. Disease associations include atopy [7], vernal keratoconjunctivitis [11], eye rubbing, hard contact lens wear [12], and noninflammatory connective tissue disorders such as Ehlers-Danlos syndrome [5]. So, the patients with noninflammatory connective tissue disease coming for an eye examination should be carefully examined for the signs of keratoconus.

There are some essays that evaluate the skin biophysical characteristics in various diseases or conditions [13–15]. In our study, we found that CRRT, stratum corneum hydration, and melanin values were significantly lower in keratoconus patients. The lower CRRT in patients with KCN is most likely due to probable alteration of elastin and collagen networks in skin along with cornea. The CRRT is influenced by collagen fibers in the upper dermis. As aging progresses, defragmentation of elastin network occurs and configuration of dermal collagen network changes, which could increase skin stiffness and decrease skin elasticity and CRRT [16]. CRRTs correlate negatively with skin stiffness and tension [10, 17]. Water is another similar ingredient in cornea and skin, so that 78% of cornea is water [1]. We found significantly lower hydration of stratum corneum in patients with KCN. This finding also put emphasis on the concurrent alterations of skin and corneal biophysical characteristics in KCN.

These changes in biophysical characteristics of skin and cornea in KCN may be related to the variations of cytokine profile in these patients. Levels of IL-6 and IL-17 were increased, while IL-12, CCL5, and IL-13 were decreased in keratoconus tear fluids compared with control [18, 19]. Another explanation of this subject is the alteration of matrix metalloproteinases in these tissues [20]. Altered activity of lysyl oxidase, a critical enzyme of the biogenesis of connective tissue, has been detected in keratoconic corneas. Lysyl oxidase is an amine oxidase that catalyzes the covalent cross-link of collagen and elastin in the extracellular environment, thus determining the mechanical properties of connective tissue. It may weaken covalent bonds between collagen and elastin fibrils, which may lead to biomechanical deterioration of the cornea [21]. These enzymes also exist in the dermis [22, 23] and it could be another explanation for skin and corneal connective tissue alteration in patients with keratoconus. Interestingly, we also find decreased elastin fibers in upper and mid dermis of three patients with keratoconus in histopathology evaluation of skin samples. These findings also reinforce the concurrent changes of skin and corneal biophysical characteristics in patients with KCN. One limitation of our study was that we could not check these cytokines or biomarkers to evaluate the possible pathogenesis of these alterations of skin and corneal biophysical characteristics in keratoconus. Another limitation was the relative small sample size of our study.

To the best of our knowledge, this study is extremely distinctive in the literature because it is the first study that evaluates the skin biophysical characteristics in patients with keratoconus and shows some variations of skin biophysical properties in these patients. However, the assessment of these skin parameters could provide us some clues to the possible common biophysical alterations of cornea and skin tissue in diseases such as keratoconus and might be helpful for better management of these patients. For example, there are some reports for estimating the risk of postsurgical ectasia in candidates for refractive surgery by establishing the diagnosis of keratoconus suspect as a contraindication for proceeding with surgery [24, 25]. The assessment of these skin parameters might provide the ophthalmologists a simple noninvasive method to detect KCN suspect especially in its early stage. So, it could be helpful to select better candidate for the refractive surgery. However, further studies with larger sample size and concurrent assessment of the related cytokines and biomarkers could be extremely more beneficial to elucidate the skin biophysical variations in patients with KCN and the possible application of these measures in clinical practice.

## Figures and Tables

**Table 1:** CRRT, stratum corneum hydration, and melanin measurements in patients with keratoconus and healthy controls.

Measurements	Patients with keratoconus (*n* = 40)	Healthy controls (*n* = 40)	*p* value
CRRT (neck)			*p* < 0.0001
Median (range)	432 (233–820)	492.5 (319–980)	
IQR	(340.5–600)	(424.8–646.5)	
CRRT (arm)			*p* < 0.0001
Median (range)	460 (228–750)	516 (427–790)	
IQR	(400.75–500)	(464.5–608.8)	
Stratum corneum hydration			*p* < 0.0001
Median (range)	30.55 (20.30–55.30)	36.60 (18.30–58.00)	
IQR	(28.08–34.15)	(32.60–40.90)	
Melanin			*p* = 0.02
Median (range)	233.5 (135–302)	238.5 (34.3–342)	
IQR	(183.25–266)	(202–271)	

CRRT, cutaneous resonance running time; IQR, interquartile range (25th–75th percentiles).

## References

[1] Komai Y., Ushiki T. (1991). The three-dimensional organization of collagen fibrils in the human cornea and sclera. *Investigative Ophthalmology and Visual Science*.

[2] Stabuc-Silih M., Strazisar M., Ravnik-Glavac M., Hawlina M., Glavac D. (2010). Genetics and clinical characteristics of keratoconus. *Acta Dermatovenerologica Alpina, Pannonica et Adriatica*.

[3] Daxer A., Fratzl P. (1997). Collagen fibril orientation in the human corneal stroma and its implication in keratoconus. *Investigative Ophthalmology & Visual Science*.

[4] Bass H. N., Sparkes R. S., Crandall B. F., Michael Marcy S. (1981). Congenital contractural arachnodactyly, keratoconus, and probable Marfan syndrome in the same pedigree. *The Journal of Pediatrics*.

[5] Rohrbach M., Vandersteen A., Yiş U. (2011). Phenotypic variability of the kyphoscoliotic type of Ehlers-Danlos syndrome (EDS VIA): clinical, molecular and biochemical delineation. *Orphanet Journal of Rare Diseases*.

[6] Kalkan Akcay E., Akcay M., Uysal B. S. (2014). Impaired corneal biomechanical properties and the prevalence of keratoconus in mitral valve prolapse. *Journal of Ophthalmology*.

[7] Bawazeer A. M., Hodge W. G., Lorimer B. (2000). Atopy and keratoconus: a multivariate analysis. *British Journal of Ophthalmology*.

[8] Agrawal V. B. (2011). Characteristics of Keratoconus patients at a tertiary eye center in India. *Journal of Ophthalmic and Vision Research*.

[9] Ruvolo E. C., Stamatas G. N., Kollias N. (2007). Skin viscoelasticity displays site- and age-dependent angular anisotropy. *Skin Pharmacology and Physiology*.

[10] Vexler A., Polyansky I., Gorodetsky R. (1999). Evaluation of skin viscoelasticity and anisotropy by measurement of speed of shear wave propagation with viscoelasticity skin analyzer. *Journal of Investigative Dermatology*.

[11] Totan Y., Hepşen I. F., Çekiç O., Gündüz A., Aydn E. (2001). Incidence of keratoconus in subjects with vernal keratoconjunctivitis: a videokeratographic study. *Ophthalmology*.

[12] McGhee C. N. J. (2009). 2008 Sir Norman McAlister gregg lecture: 150 years of practical observations on the conical cornea—what have we learned?. *Clinical and Experimental Ophthalmology*.

[13] Habig J., Vocks E., Kautzky F., Ring J. (2000). Biophysical characteristics of healthy skin and nonlesional skin in atopic dermatitis: short-term effects of ultraviolet A and B irradiation. *Skin Pharmacology and Applied Skin Physiology*.

[14] Lv C., Song S., Luo W., Elias P. M., Man M.-Q. (2012). Cutaneous resonance running time is decreased in psoriatic lesions. *Skin Research and Technology*.

[15] Seirafi H., Farsinejad K., Firooz A. (2009). Biophysical characteristics of skin in diabetes: a controlled study. *Journal of the European Academy of Dermatology and Venereology*.

[16] Xin S., Man W., Fluhr J. W., Song S., Elias P. M., Man M.-Q. (2010). Cutaneous resonance running time varies with age, body site and gender in a normal Chinese population. *Skin Research and Technology*.

[17] Quatresooz P., Hermanns J. F., Paquet P., Piérard G. E. (2006). Mechanobiology and force transduction in scars developed in darker skin types. *Skin Research and Technology*.

[18] Jun A. S., Cope L., Speck C. (2011). Subnormal cytokine profile in the tear fluid of keratoconus patients. *PLoS ONE*.

[19] Lema I., Durán J. A. (2005). Inflammatory molecules in the tears of patients with keratoconus. *Ophthalmology*.

[20] Balasubramanian S. A., Pye D. C., Willcox M. D. P. (2010). Are proteinases the reason for keratoconus?. *Current Eye Research*.

[21] Wojcik K. A., Blasiak J., Szaflik J., Szaflik J. P. (2014). Role of biochemical factors in the pathogenesis of keratoconus. *Acta Biochimica Polonica*.

[22] Rohani M. G., McMahan R. S., Razumova M. V. (2015). MMP-10 regulates collagenolytic activity of alternatively activated resident macrophages. *Journal of Investigative Dermatology*.

[23] Szauter K. M., Cao T., Boyd C. D., Csiszar K. (2005). Lysyl oxidase in development, aging and pathologies of the skin. *Pathologie Biologie*.

[24] Mcmonnies C. W. (2014). Screening for keratoconus suspects among candidates for refractive surgery. *Clinical and Experimental Optometry*.

[25] Holland S. P., Srivannaboon S., Reinstein D. Z. (2000). Avoiding serious corneal complications of laser assisted in situ keratomileusis and photorefractive keratectomy. *Ophthalmology*.

